# Supplementary figures and images for: Three-gene prognostic biomarkers for seminoma identified by weighted gene co-expression network analysis
Source: PLoS One. 2020 Oct 26;15(10):e0240943. doi: 10.1371/journal.pone.0240943 (PMC7588113; doi:10.1371/journal.pone.0240943)

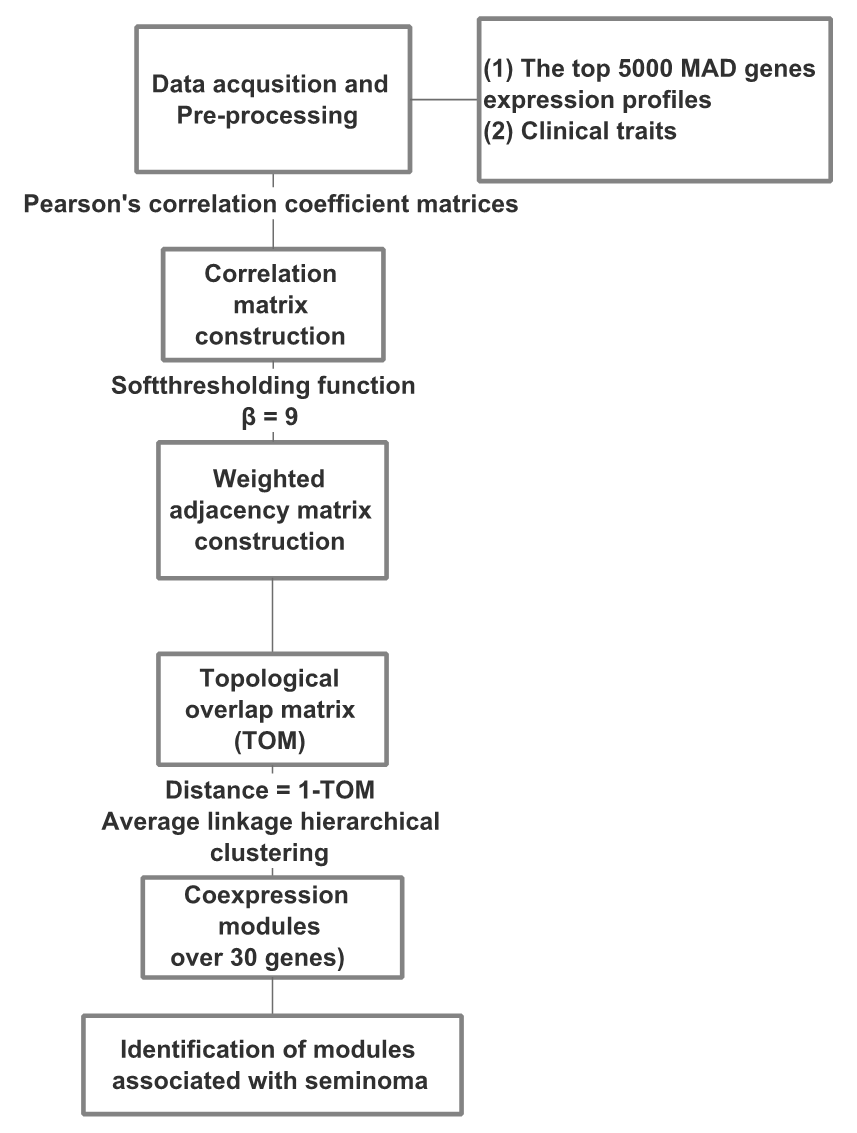

Supplement: S1 Fig — (TIF) [file pone.0240943.s001.tif]
