# Supplementary material for: Three-gene prognostic biomarkers for seminoma identified by weighted gene co-expression network analysis
Source: PLoS One. 2020 Oct 26;15(10):e0240943. doi: 10.1371/journal.pone.0240943 (PMC7588113; doi:10.1371/journal.pone.0240943)
Supplement: Attachment — Submitted filename: Rebuttal letter.docx [file pone.0240943.s002.docx]

Dear editors,

Re: Manuscript reference PONE-D-20-17839

Please find attached a revised version of our manuscript “Three-gene prognostic biomarkers for seminoma identified by weighted gene coexpression network analysis”, which we would like to resubmit for publication in PLOS ONE.

The comments of the academic editor and reviewers were highly insightful and enabled us to greatly improve the quality of our manuscript. In the following pages are our point-by-point responses to each of the comments.

Revisions in the text are shown using the red highlight for additions, and strikethrough font [example: ~~Three novel]~~ for deletions. The revisions have been organized according to the journal requirements. Written permission from the copyright holder to publish Figure 5C under the CC BY 4.0 license is obtained and uploaded simultaneously. The specific text was added to the copyrighted figure caption in the revision. We have sought for English-language editing service for help, considering many grammatical errors in the original manuscript. And the service proof is uploaded with the revisions.

We look forward to hearing from you regarding our submission. We would be glad to respond to any further questions and comments that you may have.

We shall look forward to hearing from you at your earliest convenience.

Thank you and best regards.

Yours sincerely,

Hualin Chen

Corresponding author:

Name: Gang Chen

E-mail: chengang2308@163.com

# Response to the comment of Academic Editor

1. **A related work using network modules (PMID:** **24075989) should be discussed**

Response: Thank you for your comment and this nice work has been discussed in the revision. Zaman et al performed wonderful integrated network analysis and provided a unique insight into the underlying mechanisms of cancer cell survival and proliferation driven by genomic alterations. More importantly, drug targets could be identified through this network analysis, indicating the potential significant clinical value of this work.

# Responses to the comments of Reviewer #1

1. **“Three novel prognostic biomarkers” is not clear. I suggest to revise it as “Three-gene prognostic biomarkers”.**

Response: Thanks for your advice and the title is revised.

2. **Abstract is not concise enough.**

Response: Thank you for your comment and the abstract is revised.

3. **Many grammatic errors. English should be improved.**

Response: I feel ashamed of my poor English and I am sorry for your unpleasant reading experience. I have asked the English language-editing service for help to revise the paper.

4. **Table 1: Why “P value in Survival analysis” and “P value in Univariate Cox analysis” were different? The “P value in Univariate Cox analysis” for TYROBP is not less than 0.05, and the “P value in Survival analysis” for ITGAM is also not less than 0.05. So how to interpret these genes were significantly corelated with survival?**

Response: Thanks for your comments. (1) “P-value in Survival analysis” indicated the P value calculated by log-rank test (Kaplan–Meier survival analysis), while “P-value in Univariate Cox analysis” indicated the P-value calculated by Cox proportional hazards regression model. Differences in statistical principles may cause different results. However, both methods were widely used in prognostic biomarkers identification. (2) The overall survival rate of seminoma patients was relatively high and fewer death events were observed in the TCGA datasets. If only one method was employed, some clinical valuable biomarkers may be ignored. For example, according to the KM survival analysis (log-rank test), gene *ITGAM* was not significantly correlated with prognosis. While univariate cox analysis and immunohistochemistry suggested the potential prognostic value of this biomarker. With the attempt to identify more valuable biomarkers of seminoma, we think that it may be a proper manner to integrate the results of both methods, instead of the intersection. (3) As we addressed in our paper, the lack of further experimental validation was the main drawback of our work. Therefore, further researches are urgently needed to validate our findings and explore the molecular mechanism of these biomarkers with tumor immunity in-depth.

5. **The authors claimed the three genes can be used as prognostic biomarker, but no intendent dataset was used for validation. They just analyzed the association of these genes to survival in the TCGA dataset.**

Response: I am sorry for the limitation of our work. Seminoma is rare in the general population and the health testis is much hard to obtain. Lack of tumor samples prevents us from further validation. Unfortunately, other common electronic tumor databases like the International Cancer Genome Consortium (ICGC) do not provide seminoma cases for analysis. However, the three biomarkers were also DEGs in GSE8607.

I appreciate your great efforts in our work and your precious comments help us improve the quality of our manuscript.

# Responses to the comments of Reviewer #2

1. **Please clearly state the major innovation of this work.**

Response: (1) This is the first study using WGCNA to identify prognostic biomarkers of seminoma and three prognostic biomarkers, which may be potential immunotherapy targets, are obtained; (2) Tumor immunity is a hot topic currently and previous studies have mentioned the critical role of the immune response in seminoma patients. Our findings further prove the roles of tumor immune in seminoma, which may provide evidence for future immunotherapy.

2. **More details about the construction of weighted correlation network should be given. How was the correlation matrix constructed and transformed into weighted adjacency matrix? What is the blue and green modules?**

Response: Thanks for your suggestions. The official tutorials (available at <https://horvath.genetics.ucla.edu/html/CoexpressionNetwork/Rpackages/WGCNA/>) provide the codes for analysis in detail. What we have to do is to adjust some thresholds according to our samples. (1) More details about the construction of the weighted correlation network are given in the revision. (2) The correlation matrix is constructed based on Pearson correlation analysis (more details are given in the revision). Then, a soft‑thresholding function is used to transform the correlation matrix into a weighted and scale-free adjacency matrix. In this part, identification of a soft-thresholding β (a soft-thresholding parameter enhancing strong correlations between genes and penalizing weak correlations) is of importance. (3) The blue and green modules represent different modules, and the colors of the modules are just used to mark and distinguish these different modules. The colors have no relation to the genes. Genes in the same module have strong correlation with each.

3. **Please add a flowchart of the construction of weighted correlation network.**

Response: Thank you for your advice. Actually, Figure 2 A-E represents the main workflow of the whole WGCNA. However, we generate a flowchart of the construction of a weighted correlation network according to your suggestion and it is uploaded as a supplementary figure.

4. **Please give the reason for parameter selection (e.g., the soft threshold).**

Response: Thanks for your comment. (1) Soft threshold: a suitable soft threshold is determined by two factors: scale-independence and connectivity. In our study, scale-independence >0.85 with maximum average connectivity is considered as the criteria for soft threshold selection. And as Figure 2A demonstrated, only power 9 meets the criteria and is selected for further analysis. As reported in the official guidelines, scale-independence >0.80 is acceptable in soft threshold selection. Under certain special conditions, it is less likely to obtain proper power. And power 6 is often empirically used. (2) Adjust P-value < 0.01 and |logFC| ≥2: Actually, both 0.01 and 0.05 can be used as the thresholds of Adjust P-value. As for |logFC|, 1, 2 or even 0.5 is acceptable. There is no strict restriction in the thresholds of Adjust P-value and |logFC|. In our study, the reason we choose a stricter threshold is that a large number of DEGs in GSE8607 are identified through initial analysis and we only want to find out more significant genes with lower Adjust P-value and higher |logFC|.

5. **I want to know whether this network analysis can also be used for the analysis of the other cancers?**

Response: Thanks for your question and the answer is yes.

6. **You should revise your English writing carefully and eliminate small errors in the paper to make the paper easier to understand.**

Response: I am extremely sorry for the grammar errors in our original paper. I have sought for language-editing service for help.

7. **Could you discuss the recent trend of developing** **computational model for identification of the non-coding RNA biomarker of human complex diseases as the future direction of your current research about biomarker identification of seminoma? Some important studies should be cited and discussed (PMIDs:** **31927572,** **29939227,** **29045685, and** **24002109).**

Response: Your comments and advice are filled with wisdom. These researches contribute a lot to the development of computational biology and these computational models provide new methods to find novel and more significant disease-related non-coding RNA biomarkers. More excellent works, I believe, could be carried out soon based on these models. These works have been cited and discussed in the revision.
